# Persistence of Hyper-Ramified Microglia in Porcine Cortical Gray Matter after Mild Traumatic Brain Injury

**DOI:** 10.3390/biomedicines11071960

**Published:** 2023-07-12

**Authors:** Michael R. Grovola, Alan Jinich, Nicholas Paleologos, Edgardo J. Arroyo, Kevin D. Browne, Randel L. Swanson, John E. Duda, D. Kacy Cullen

**Affiliations:** 1 Center for Neurotrauma, Neurodegeneration & Restoration, Corporal Michael J. Crescenz VA Medical Center, Philadelphia, PA 19104, USA; mgrovola@pennmedicine.upenn.edu (M.R.G.); ajinich@sas.upenn.edu (A.J.); npale@sas.upenn.edu (N.P.); arroyoe@pennmedicine.upenn.edu (E.J.A.); kbrowne@pennmedicine.upenn.edu (K.D.B.); randel.swanson@pennmedicine.upenn.edu (R.L.S.); 2 Center for Brain Injury & Repair, University of Pennsylvania, Philadelphia, PA 19104, USA; 3 Department of Physical Medicine and Rehabilitation, Perelman School of Medicine, University of Pennsylvania, Philadelphia, PA 19104, USA; 4 Department of Neurology, Perelman School of Medicine, University of Pennsylvania, Philadelphia, PA 19104, USA; 5 Parkinson’s Disease Research, Education and Clinical Center, Corporal Michael J. Crescenz VA Medical Center, Philadelphia, PA 19104, USA; 6 Department of Bioengineering, School of Engineering and Applied Science, University of Pennsylvania, Philadelphia, PA 19104, USA

**Keywords:** mild TBI, neuroinflammation, microglia, large animal models, fibrinogen

## Abstract

Traumatic brain injury (TBI) is a major contributor to morbidity and mortality in the United States as several million people visit the emergency department every year due to TBI exposures. Unfortunately, there is still no consensus on the pathology underlying mild TBI, the most common severity sub-type of TBI. Previous preclinical and post-mortem human studies have detailed the presence of diffuse axonal injury following TBI, suggesting that white matter pathology is the predominant pathology of diffuse brain injury. However, the inertial loading produced by TBI results in strain fields in both gray and white matter. In order to further characterize gray matter pathology in mild TBI, our lab used a pig model (n = 25) of closed-head rotational acceleration-induced TBI to evaluate blood-brain barrier disruptions, neurodegeneration, astrogliosis, and microglial reactivity in the cerebral cortex out to 1 year post-injury. Immunohistochemical staining revealed the presence of a hyper-ramified microglial phenotype—more branches, junctions, endpoints, and longer summed process length—at 30 days post injury (DPI) out to 1 year post injury in the cingulate gyrus (*p* < 0.05), and at acute and subacute timepoints in the inferior temporal gyrus (*p* < 0.05). Interestingly, we did not find neuronal loss or astroglial reactivity paired with these chronic microglia changes. However, we observed an increase in fibrinogen reactivity—a measure of blood-brain barrier disruption—predominately in the gray matter at 3 DPI (*p* = 0.0003) which resolved to sham levels by 7 DPI out to chronic timepoints. Future studies should employ gene expression assays, neuroimaging, and behavioral assays to elucidate the effects of these hyper-ramified microglia, particularly related to neuroplasticity and responses to potential subsequent insults. Further understanding of the brain’s inflammatory activity after mild TBI will hopefully provide understanding of pathophysiology that translates to clinical treatment for TBI.

## 1. Introduction

Traumatic brain injury (TBI) is a major contributor to morbidity and mortality in the United States. According to national TBI incidence reports, several million people visit the emergency department each year with unintentional falls, being unintentionally stuck by or against an object, and motor vehicle crashes as the most common mechanisms of injury [1]. Mild TBI, also known as concussion, is much more common than moderate or severe TBI, with the incidence of TBI highest in males among the adult population [2]. Unfortunately, there is still no consensus on the pathology underlying mild TBI as mild TBI is partly defined by an absence of structural changes on clinical neuroimaging, such as hematoma, contusion, and brain swelling [3].

In order to further characterize mild TBI pathology, our lab uses a pig model of closed-head rotational acceleration-induced TBI. This large animal model emulates human TBI by providing more representative biomechanical loading conditions, brain anatomy, and neurophysiology compared to rodent models (see [4] for a review). Previous studies in this porcine model of TBI, as well as post-mortem human TBI studies, have detailed the presence of diffuse axonal injury, suggesting that white matter pathology is the predominant pathology of diffuse brain injury [5,6,7,8,9,10,11]. Yet recent neuroimaging studies suggest that gray matter atrophy occurs at acute and chronic timepoints after mild TBI in humans [12,13,14].

Indeed, the inertial loading produced by TBI results in strain fields in both gray and white matter. One of the consequences of this loading is transient disruption of the neuronal plasma membrane [15,16]. Permeabilized membranes can trigger disruption of normal cell function in a positive feedback manner based on loss of membrane charge, interruption of electrokinetic transport, and osmotic imbalance [17]. Studies suggest that many initially permeabilized cells survive the insult; however, there may be prolonged alteration in physiology or later cell death [18,19,20]. Previous in vitro and rodent studies suggest that neuronal membrane disruption may occur in separate waves following TBI [21,22]. Moreover, our research team has discovered membrane disruptions in cortical neuron somata paired with significant microglial alterations after TBI [23]. This gray matter pathology may play a pivotal role in post-TBI dysfunction, yet cortical pathology has not been thoroughly characterized in closed-head diffuse TBI.

Here, we sought to evaluate trauma-induced changes to microglia, astrocytes, neurons, and the blood-brain barrier (BBB) within the cerebral cortex using an established large-animal model of closed-head diffuse TBI. We assessed neuroinflammatory and neurodegenerative changes in the cerebral cortex up to 1 year post-injury, with a particular focus on the cingulate gyrus and inferior temporal gyrus. We examined disparate areas of the cortex—one medial gyrus and one lateral gyrus—that may be subjected to variable inertial loading and therefore different pathological patterns and distributions. Specifically, we performed detailed quantification of subtle yet discrete microglial morphological features through skeletal analysis in order to characterize the neuroimmune response to TBI in the cortex. To provide context for these detailed microglial analyses, we also assessed astrocyte reactivity by morphological and cell density measures, cortical neuron dystrophy or loss via cell density measures, and BBB disruption through the presence of blood proteins in the brain parenchyma. This work contributes to a growing body of literature suggesting that TBI-induced gray matter pathology may drive additional neuroinflammatory and neurodegenerative sequela. We hypothesized that microglial morphological changes would coincide with astroglial reactivity, neuronal dystrophy/loss, and BBB disruption in the cerebral cortex after TBI. Understanding these often-overlooked gray matter biophysical responses is crucial to the development of injury prevention strategies and therapeutic interventions following TBI.

## 2. Materials and Methods

### 2.1. Animal Subjects

All procedures were approved by the Institutional Animal Care and Use Committees at the University of Pennsylvania and the Michael J. Crescenz Veterans Affairs Medical Center and adhered to the guidelines set forth in the NIH Public Health Service Policy on Humane Care and Use of Laboratory Animals (2015).

For the current study, specimens were obtained from a tissue archive of castrated male pigs subjected to a single mild TBI. This tissue archive was also used in Grovola et al. [9,24]. All pigs were 5–6 months old, sexually mature (considered to be young adult), Yucatan miniature pigs at a mean weight of 34 ± 4 kg (total n = 25, mean ± SD). Pigs were fasted for 16 h, then anesthesia was induced with 20 mg/kg of ketamine and 0.5 mg/kg of midazolam. Following induction, 0.1 mg/kg of glycopyrrolate was subcutaneously administered and 50 mg/kg of acetaminophen was administered per rectum. All animals were intubated with an endotracheal tube and anesthesia was maintained with 2% isoflurane per 2 L O_2_. Heart rate, respiratory rate, arterial oxygen saturation, and temperature were continuously monitored throughout the experiment. A forced-air temperature management system was used to maintain normothermia throughout the procedure.

### 2.2. Head Rotational TBI

In order to attain closed-head diffuse mild TBI in animals, we used a previously described model of head-rotational acceleration in pigs [4,6]. Similar methods were described in Grovola et al. [24]. Briefly, each animal’s head was secured to a bite plate, which itself was attached to a pneumatic actuator and a custom assembly that converts linear motion into angular momentum. The pneumatic actuator rotated each animal’s head in the coronal plane, reaching an angular velocity between 230–270 rad/sec (n = 15). Any presence of apnea was recorded (maximum apnea time = 45 s), and animals were hemodynamically stabilized if necessary. No animals were excluded from the study due to apnea or hemodynamic instability. Sham animals (n = 10) underwent identical protocols, including being secured to the bite plate, however the pneumatic actuator was not initiated. All animals were transported back to their housing facility, monitored acutely for 3 h, and given access to food and water. Afterwards, the animals were monitored daily for 3 days by veterinary staff.

### 2.3. Specimen Preparation

At 3 days post-injury (DPI) (n = 4), 7 DPI (n = 5), 30 DPI (n = 3), or 1 year post-injury (YPI) (n = 3), animals were induced and intubated as described above. Sham animals survived for 7 days (n = 4), 30 days (n = 1), or 1 year (n = 5). While under anesthesia, animals were transcardially perfused with 0.9% heparinized saline followed by 10% neutral buffered formalin (NBF). Animals were then decapitated, and tissue stored overnight in 10% NBF at 4 °C. The following day, the brain was extracted, weighed, and post-fixed in 10% NBF at 4 °C for one week. To block the tissue, an initial coronal slice was made immediately rostral to the optic chiasm. The brain was then blocked into 5 mm thick coronal sections from that point by the same investigator. This allowed for consistent blocking and section coordinates across animals. All blocks of tissue were paraffin embedded and 8 µm thick sections were obtained using a rotary microtome. Two sections from each pig—one containing anterior aspects of hippocampal tissue (approximately 10 mm posterior to the optic chiasm) and one containing posterior aspects of hippocampal tissue (approximately 15 mm posterior to the optic chiasm)—were used for histological analysis. Of note, one of the enrolled specimens in the 3 DPI group was excluded from the current study due to an unresolvable error in tissue processing resulting in low-quality and inconsistent tissue staining.

### 2.4. Immunohistochemical Staining and Microscopy

For 3,3′-Diaminobenzidine (DAB) immunohistochemical labeling, we used a protocol outlined in Johnson et al. [25]. Briefly, slides were dewaxed in xylene, rehydrated in ethanol and de-ionized water. Antigen retrieval was completed in Tris EDTA buffer pH 8.0 using a microwave pressure cooker then blocked with normal horse serum. Slides were incubated overnight at 4 °C using either an anti-rabbit fibrinogen (abcam, Waltham, MA, USA, 183109, 1:5000), an anti-mouse GFAP (SMI-22) (MilliporeSigma, Burlington, MA, USA, NE1015, 1:12,000), or an anti-rabbit Iba-1 (Wako Chemicals, Richmond, VA, USA, 019-19741, 1:4000) primary antibody. The following day, slides were rinsed in PBS and incubated in a horse anti-mouse/rabbit biotinylated IgG secondary antibody (VECTASTAIN Elite ABC Kit, Vector Laboratories, Newark, CA, USA, PK-6200). Sections were rinsed again, then incubated with an avidin/biotinylated enzyme complex (VECTASTAIN Elite ABC Kit), rinsed again, and incubated with the DAB enzyme substrate (Vector Laboratories, SK-4100) for 7 min. Sections were counterstained with hematoxylin, dehydrated in ethanol, cleared in xylene, and finally coverslipped using cytoseal. All sections were stained in the same histological sample run.

For Cresyl Violet (CV) staining, slides were dewaxed in xylene, and rehydrated in ethanol and deionized water. Slides were placed in a solution of 0.1% cresyl violet acetate (MilliporeSigma, C5042) and deionized water for 5 min, rinsed in deionized water, and then differentiated in an acetic acid and 95% ethanol solution. Slides were dehydrated in ethanol, cleared in xylene, and finally coverslipped using cytoseal. All slides were stained in the same histological sample run.

For fluorescence immunohistochemical staining, slides were dewaxed in xylene, and rehydrated in ethanol and deionized water. Antigen retrieval was completed in Tris EDTA buffer pH 8.0 using a microwave pressure cooker then blocked with normal horse serum. Slides were incubated overnight at 4 °C using anti-mouse Neuronal Nuclei (NeuN) (MilliporeSigma, MAB377, 1:500) primary antibody. The following day, sections were rinsed in PBS and incubated in donkey anti-mouse 555 (Thermo Fisher Scientific, Waltham, MA, A31570, 1:500) secondary antibody for 60 min. Sections were rinsed again, then incubated with Hoechst (Thermo Fisher Scientific, H3570, 1:10,000) to visualize DNA, and finally cover slipped using Fluoromount-G (Southern Biotech, Birmingham, AL, USA, 0100-01). All sections were stained on the same histological sample run.

All GFAP, Iba-1, and CV sections were imaged and analyzed at 20× optical zoom using an Aperio CS2 digital slide scanner (Leica Biosystems Inc., Buffalo Grove, IL, USA). All Fibrinogen sections were imaged at 100× using a Keyence VHX-6000 digital microscope (Itasca, IL, USA). All NeuN sections were imaged at 10× using a Keyence BZ-X7000 digital microscope (Itasca, IL, USA).

### 2.5. Neuropathological Analysis

For Iba-1 skeletal analysis, we employed methods from Grovola et al. [9]. Briefly, we imaged five 40× images per anatomical region for analysis. To conduct skeletal analysis, all Iba-1 positive cells in each 40× field were manually selected, and the image was deconvoluted using Fiji software (version 2.9.0, National Institute of Health, Bethesda, MD, USA). Bandpass filters, unsharp mask, and close plugins were applied before converting the image to binary, skeletonizing, and removing skeletons not overlaid with the manually selected cells. The Analyze Skeleton plugin was then applied to quantify skeletal features such as number of process branches, junctions, process endpoints, and slab voxels in order to measure changes in microglia ramification [26]. For each image, each feature was summed then divided by the total number of cells, thus providing a single field average normalized per cell. Therefore, we examined 5 values from 5 images in the same histological slide for each animal in each anatomical region, which serves as a repeated measure, regional analysis. Slab voxels were then multiplied by the volume of the voxel to calculate the summed process length per cell.

For CV cell counts, a single 20× photomicrograph was taken in layer 2/3 at the gyri apex of the cingulate gyrus for each CV-stained slide. Total cell density was assessed using Fiji software (National Institute of Health); images were converted to grayscale and the Analyze Particles plugin was used to count cells in an automated fashion using an objective set of exclusion parameters [27]. Total neuron density was determined by manual counting; neurons were distinguished from glia by morphology, staining pattern, and sometimes size using the guidelines set by Garcia-Cabezas et al. [28]. All counted neurons had a visible nucleus, and a small rim of visible cytoplasm around the nucleus to distinguish small neurons from astrocytes. All cell densities were normalized according to tissue area. The cell densities were averaged for each specimen in lieu of traditional stereology.

For NeuN neuron counts, the entire left hemisphere cingulate gyrus was stitched at 10× resolution for each specimen. Each image was then cropped to include either cortical layers II/III or all cortical gray matter layers. We assessed total cell density using Fiji software (National Institute of Health); images were converted to grayscale and the Analyze Particles plugin was used to count cells in an automated fashion using an objective set of exclusion parameters [27]. All cell densities were normalized according to tissue area. Neuron density was averaged for each specimen in lieu of traditional stereology.

For astrocyte semi-quantitative analysis, inferior temporal gyrus and cingulate gyrus were assessed in 2 sections per specimen. We adapted a semi-quantitative scale from Sofroniew et al. to histologically classify the progressive severity of reactive astrocytes [29]. Each region was given a 0–3 glial fibrillary acidic protein (GFAP) reactivity score based on cell body size, upregulation of GFAP, and density of GFAP+ cells in the region.

For fibrinogen analysis, we determined percentage area of fibrinogen coverage via the Cavalieri estimator probe within Stereo Investigator software (version 11.06.01, MBF Bioscience, Williston, VT, USA). This probe created a point grid over the tissue and the sum of the number of points that hit fibrinogen-stained regions of interest were used to estimate the area. This fibrinogen area was then divided by the total brain parenchyma area to provide a percentage area of fibrinogen reactivity. Percentage area was averaged between the 2 sections per specimen to provide a more global assessment of fibrinogen reactivity. Different markers were used to label gray versus white matter to allow for comparative analysis in different types of neuronal tissue. Therefore, we assessed total brain, gray matter, and white matter for percentage area fibrinogen reactivity.

### 2.6. Statistical Analysis

Statistical analysis was performed using GraphPad Prism statistical software (version 9.5.1, GraphPad Software Inc. La Jolla, CA, USA). Any outliers were removed using the ROUT method (Q = 1%). Due to low sample size, GFAP reactivity, CV cell counts, NeuN cell counts, and total fibrinogen reactivity were analyzed with a Kruskal–Wallis test and Dunn’s multiple comparisons. Kruskal–Wallis test results are reported as (*H* (degrees of freedom) = *H* test statistic, *p*-value). A T-test was used to analyze gray versus white matter fibrinogen reactivity and reported as (t (degrees of freedom) = t statistic, p-value). The skeletal analysis was statistically assessed via One-way analysis of variance (ANOVA) and Tukey’s multiple comparisons test. One-way ANOVA results are reported as (*F* (degrees of freedom numerator, degrees of freedom denominator) = *F* value, *p*-value). Mean, median, standard deviation, and 95% confidence intervals were reported. Differences were considered significant if *p* < 0.05. As this was an archival study, power calculations were not used to determine the number of specimens for each experimental group. The number of images chosen for skeletal analysis mirrors our work from a previous study [9].

## 3. Results

### 3.1. Microglia Skeletal Analysis Revealed Chronic Changes in the Cingulate Gyrus

Our previous research has shown that microglia alter their morphology in the white matter in response to a single mild TBI [9]. Therefore, we sought to quantify microglia morphological changes in gray matter structures via automated skeletal analysis. We exclusively analyzed layers II/III of the cerebral cortex in the cingulate gyrus as neurons are preferentially damaged in these cortical layers after mild TBI in our model of injury [30].

**Figure 1:**
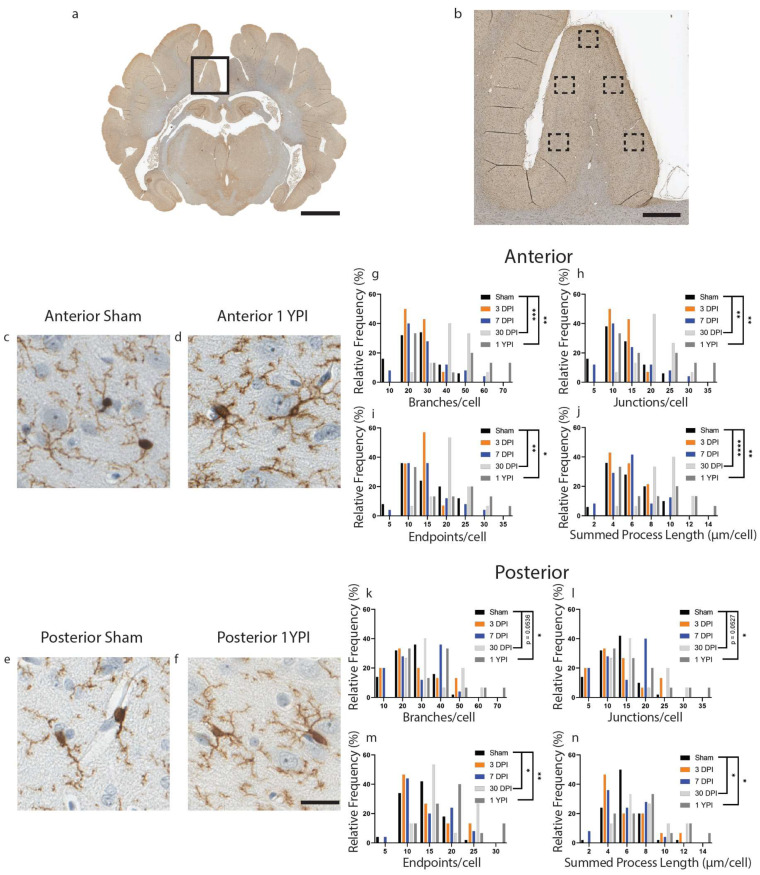
Microglia Become Hyper-ramified in the Cingulate Gyrus at One Month and Persist out to Chronic Time Points. A whole coronal section of Iba-1-stained tissue is shown with a call-out box centered around the cingulate gyrus (**a**, scale = 7 mm). Five call-out boxes in the cingulate gyrus depict the location of the 40× images (image n = 5 per animal) used for skeletal analysis (**b**, scale = 1 mm). Cropped 40× images for sham and 1 YPI are displayed showing the hyper-ramified morphology at 1 YPI (**c**–**f**, scale = 30 µm). Histograms display the range of data for each experimental group. There is a significant increase in the number of branches, junctions, process endpoints, and summed process length at 30 DPI and 1 YPI compared to sham in anterior sections of cingulate gyrus (**g**–**j**) and posterior sections of cingulate gyrus (**k**–**n**) (* *p* < 0.05, ** *p* ≤ 0.01, *** *p* ≤ 0.001, **** *p* ≤ 0.0001).

There was a significant increase in the number of branches, junctions, endpoints, and summed process length per microglia at 30 DPI and 1 YPI in the anterior cingulate gyrus compared to age-matched sham (Figure 1) (Table 1). There were no statistically significant differences between any of the sham timepoints out to 1 YPI. There was an increase in the number of branches per microglia (*F* (4114) = 7.199, *p* < 0.0001) at 30 DPI (*p* = 0.0006) and 1 YPI (*p* = 0.0023) compared to sham (Figure 1g). There was an increase in the number of junctions per microglia (*F* (4114) = 7.235, *p* < 0.0001) at 30 DPI (*p* = 0.0006) and 1 YPI (*p* = 0.0022) compared to sham (Figure 1h). There was an increase in the number of endpoints per microglia (*F* (4114) = 5.358, *p* = 0.0005) at 30 DPI (*p* = 0.0043) and 1 YPI (*p* = 0.0256) compared to sham (Figure 1i). Finally, there was an increase in the summed process length per microglia (*F* (4113) = 8.825, *p* < 0.0001) at 30 DPI (*p* < 0.0001) and 1 YPI (*p* = 0.0062) compared to sham (Figure 1j).

In the posterior cingulate gyrus, there was an increase in the number of branches, junctions, endpoints, and summed process length per microglia at 30 DPI and 1 YPI compared to age-matched sham (Figure 1) (Table 2). There were no statistically significant differences between any of the sham timepoints out to 1 YPI. The number of branches per microglia increased (*F* (4115) = 3.932, *p* = 0.0050) at 30 DPI (*p* = 0.0536) and 1 YPI (*p* = 0.0147) compared to sham (Figure 1k). The number of junctions increased (*F* (4115) = 3.830, *p* = 0.0058) at 30 DPI (*p* = 0.0527) and 1 YPI (*p* = 0.0177) compared to sham (Figure 1l). The number of endpoints increased (*F* (4115) = 4.261, *p* = 0.0030) at 30 DPI (*p* = 0.0489) and 1 YPI (*p* = 0.0098) compared to sham (Figure 1m). Finally, the summed process length per microglia increased (*F* (4115) = 4.359, *p* = 0.0026) at 30 DPI (*p* = 0.0369) and 1 YPI (*p* = 0.0282) compared to sham (Figure 1n). These data detail a hyper-ramified microglial morphology at 30 DPI and 1 YPI in both anterior and posterior sections of the cingulate gyrus.

### 3.2. Microglia Skeletal Analysis Revealed Acute and Subacute Changes in the Inferior Temporal Gyrus

**Figure 2:**
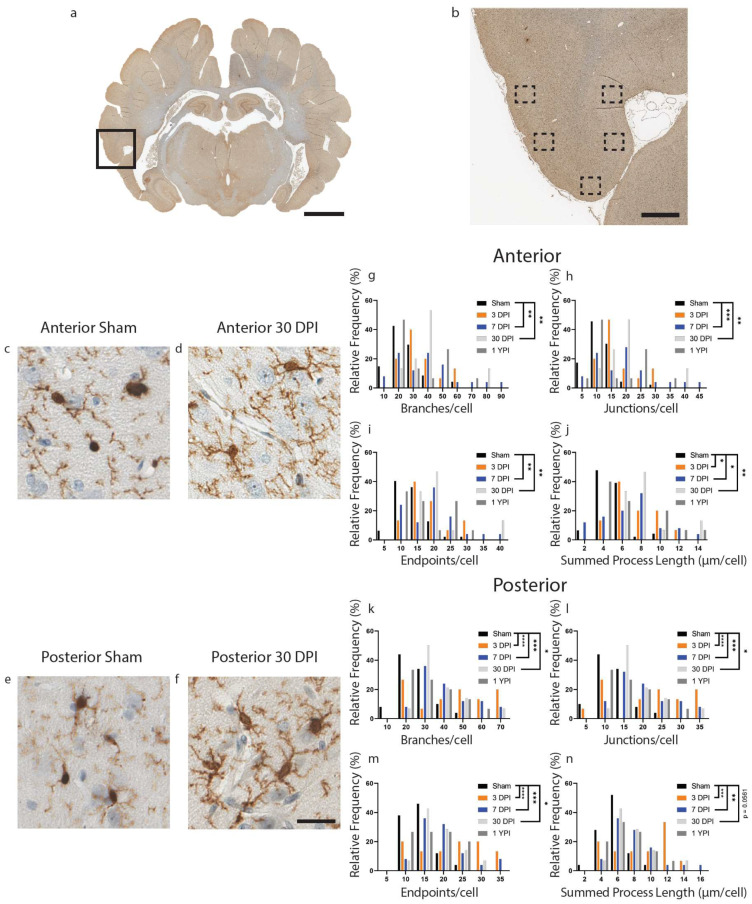
Microglia Become Hyper-ramified in the Inferior Temporal Gyrus at Acute and Subacute Time Points. A whole coronal section of Iba-1-stained tissue is shown with a call-out box centered around the inferior temporal gyrus (**a**, scale = 7 mm). Five call-out boxes in the inferior temporal gyrus depict the location of the 40× images (image n = 5 per animal) used for skeletal analysis (**b**, scale = 1 mm). Cropped 40× images for sham and 30 DPI are displayed showing the comparatively hyper-ramified morphology at 30 DPI in posterior tissue (**c**–**f**, scale = 30 µm). Histograms display the range of data for each experimental group There is a significant increase in the number of branches (**g**), junctions (**h**), and process endpoints (**i**) at 7 DPI and 30 DPI, as well as a significant increase in summed process length (**j**) at 3 DPI, 7 DPI and 30 DPI in anterior sections of inferior temporal gyrus. There is a significant increase in the number of branches (**k**), junctions (**l**), process endpoints (**m**), and summed process length (**n**) at 3 DPI, 7 DPI, and 30 DPI in posterior sections of inferior temporal gyrus (* *p* < 0.05, ** *p* ≤ 0.01, *** *p* ≤ 0.001, **** *p* ≤ 0.0001).

Next, we analyzed microglial morphological changes in layers II/III of the inferior temporal gyrus, a lateral cortical structure. In the inferior temporal gyrus, there were significant changes in microglia morphology detected in anterior sections of tissue compared to sham (Figure 2) (Table 3). Specifically, we found an increase in the number of branches, junctions, and endpoints at 7 DPI and 30 DPI, as well as an increase in summed process length per microglia at 3 DPI, 7 DPI, and 30 DPI. The number of branches per microglia increased (*F* (4112) = 5.697, *p* = 0.0003) at 7 DPI (*p* = 0.0015) and 30 DPI (*p* = 0.0040) compared to sham (Figure 2g). The number of junctions per microglia increased (*F* (4111) = 6.468, *p* = 0.0001) at 7 DPI (*p* = 0.0006) and 30 DPI (*p* = 0.0018) compared to sham (Figure 2h). The number of endpoints per microglia increased (*F* (4112) = 6.053, *p* = 0.0002) at 7 DPI (*p* = 0.0011) and 30 DPI (*p* = 0.0023) compared to sham (Figure 2i). Finally, the summed process length per microglia increased (*F* (4111) = 5.788, *p* = 0.0003) at 3 DPI (*p* = 0.0272), 7 DPI (*p* = 0.0140) and 30 (*p* = 0.0016) compared to sham (Figure 2j).

Importantly, we did find a significant difference between sham specimens which survived for 30 days or less and sham specimens which survived out to 1 year. Specifically, we found a decrease in the number of branches (t (45) = 5.641, *p* < 0.0001), junctions (t (44) = 5.775, *p* < 0.0001), endpoints (t (45) = 5.965, *p* < 0.0001), and summed process length (t (44) = 4.462, *p* < 0.0001) in sham which survived out to one year compared to sham which survived for 30 days or less.

There were also significant differences in branches, junctions, endpoints, and summed process length in posterior sections of tissue at acute and subacute timepoints (Figure 2) (Table 4). The number of branches per microglia increased (*F* (4,114) = 9.397, *p* < 0.0001) at 3 DPI (*p* < 0.0001), at 7 DPI (*p* = 0.0001), and 30 DPI (*p* = 0.0293) compared to sham (Figure 2k). The number of junctions increased (*F* (4114) = 9.323, *p* < 0.0001) at 3 DPI (*p* < 0.0001), at 7 DPI (p = 0.0001), and 30 DPI (p = 0.0290) compared to sham (Figure 2l). The number of endpoints increased (*F* (4114) = 9.458, *p* < 0.0001) at 3 DPI (*p* < 0.0001), at 7 DPI (*p* = 0.0002), and 30 DPI (*p* = 0.0346) compared to sham (Figure 2m). Finally, the summed process length per microglia increased (*F* (4114) = 7.580, *p* < 0.0001) at 3 DPI (*p* = 0.0001), at 7 DPI (*p* = 0.0013) and 30 DPI (*p* = 0.0561) compared to sham (Figure 2n). Overall, we found hyper-ramified microglial morphology at 3, 7 and 30 DPI in both anterior and posterior sections of the inferior temporal gyrus.

Finally, we did find a significant difference between sham specimens which survived for 30 days or less and sham specimens which survived out to 1 year. Specifically, we found a decrease in the number of branches (t (48) = 2.724, *p* = 0.0090), junctions (t (48) = 2.661, *p* = 0.0106), endpoints (t (48) = 2.842, *p* = 0.0066), and summed process length (t (48) = 2.129, *p* = 0384) in sham which survived out to one year compared to sham which survived for 30 days or less.

### 3.3. Total Cell Density and Neuron Density Did Not Change in the Cingulate Gyrus after Mild TBI

**Figure 3:**
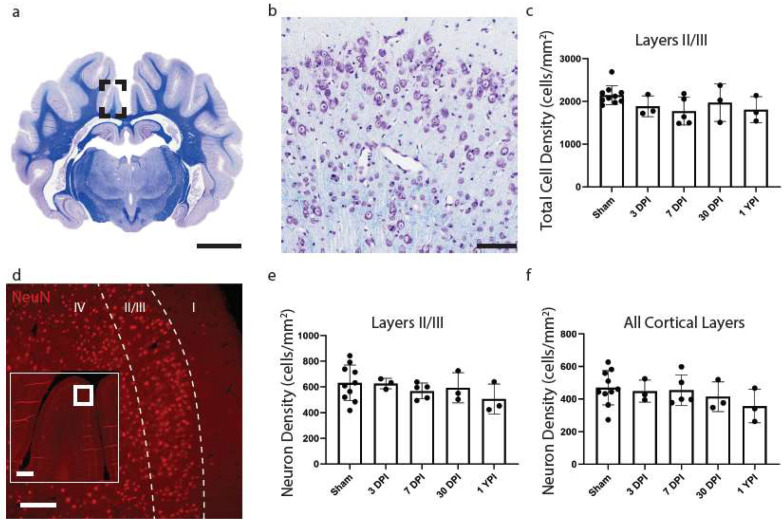
Total Cell Density and Neuronal Density Does Not Change in the Cingulate Gyrus. A whole coronal section of Luxol Fast Blue and Cresyl Violet stained tissue is shown with a call out box centered around the apex of the cingulate gyrus (**a**, scale = 7 mm). A 20× image of cortical cells is shown (**b**, scale = 100 µm). There were no significant changes to total cell density (**c**) as a result of TBI. Whole coronal sections of tissue were also stained with NeuN, a neuronal marker, to count neurons in layers II/III and all cortical layers across the cingulate gyrus. A 10× image of NeuN is shown denoting cortical layers II/III (**d**, scale = 200 µm) with an inset image demonstrating its location along the cingulate gyrus (scale = 500 µm). There were no significant changes in neuron density in layers II/III (**e**) or among all cortical layer (**f**). Each point represents data from an individual animal within each group.

As the cingulate gyrus had chronic microglial morphological changes, consistent changes between anterior and posterior aspects of tissue, and no significant differences between aged matched shams, additional pathological analyses focused on this region. Total cell density was next calculated to evaluate any overt glial cell proliferation or loss. However, there were no significant changes to CV total cell density (*H* (4) = 5.246, *p* = 0.2630) (Figure 3c). Cohen’s *d* effect sizes are reported below to demonstrate the magnitude of difference between sham and experimental groups and allow for *a priori* power analysis (Table 5).

To assess potential neuronal loss in layers II/III compared to all cortical layers of the cingulate gyrus, we conducted NeuN cell counts in tissue sections adjacent to CV-stained sections. However, there were no significant changes to neuron density in layers II/III (*H* (4) = 2.699, *p* = 0.6093) (Figure 3e) or all cortical layers (*H* (4) = 3.469, *p* = 0.4826) (Figure 3f of the cingulate gyrus.

### 3.4. GFAP Reactivity Did Not Change after Single Mild TBI

To further explore the extent of gray matter pathology after mild TBI and to complement our previous pathological analysis in the white matter, we chose two gray matter structures for closer assessment: the cingulate gyrus, a midline structure, and the inferior temporal gyrus, a lateral structure. We chose these structures based on previous studies in our lab that described permeabilized gray matter neurons after closed-head rotational mild TBI; patches of permeabilized cells were seen in some gyri but absent in others [30]. This pathological distribution suggests that post-TBI gray matter pathology is multi-focal yet may skip some anatomical levels of the cortex. Examination of the cingulate and inferior temporal gyri allows us to thoroughly examine two sample cortices for pathological sequela to TBI.

**Figure 4:**
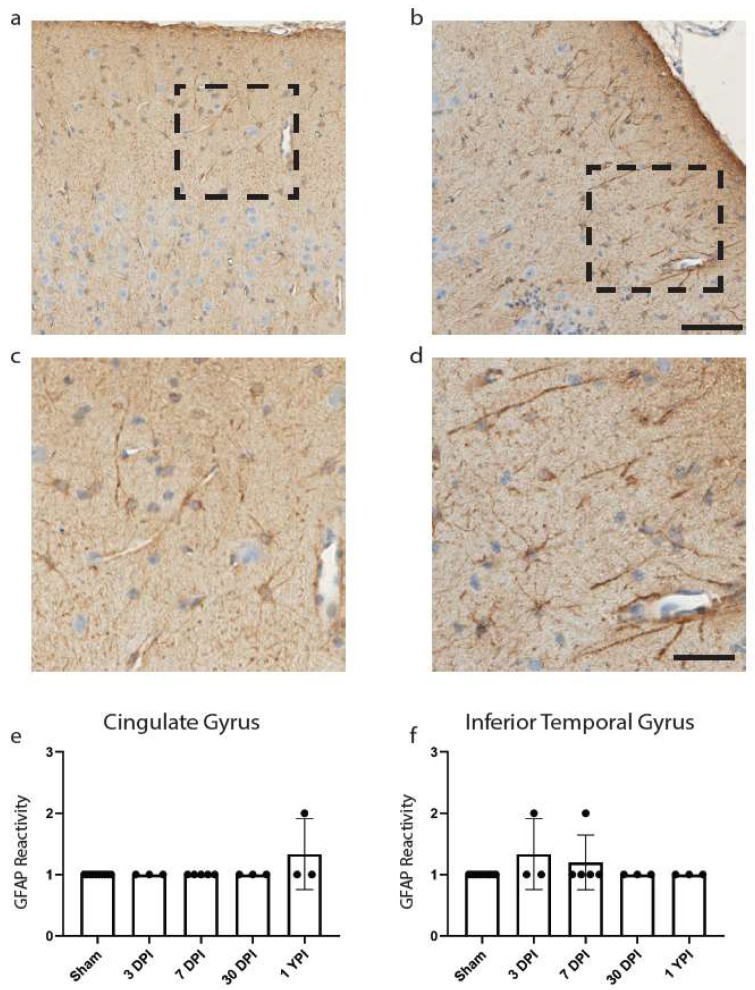
GFAP Reactivity Does Not Change in the Cingulate or Inferior Temporal Gyrus. Examples of GFAP reactivity scored as a 1 (**a**) or 2 (**b**) are shown (scale = 100 µm) with corresponding call-out boxes for scores of 1 (**c**) or 2 (**d**; scale = 50 µm). No images were scored as a 3. There was no significant change in GFAP reactivity in cingulate gyrus (**e**) or inferior temporal gyrus (**f**). Each point represents data from an individual animal within each group.

We began with astrocytic changes as measured by GFAP reactivity as astrocytic end-feet are a component of the BBB, which we have noted was disrupted in our fibrinogen reactivity analysis below. Reactive astrogliosis is often used as a marker for damaged CNS tissue and has been observed colocalizing with fibrinogen blood proteins [29,31]. Yet there was no significant change in GFAP reactivity in the cingulate gyrus (*H* (4) = 7.00, *p* = 0.1359) (Figure 4e), nor was there a change in GFAP reactivity in the inferior temporal gyrus (*H* (4) = 4.60, *p* = 0.3309) (Figure 4f).

### 3.5. Fibrinogen Reactivity Increased at 3 DPI after Mild TBI

**Figure 5:**
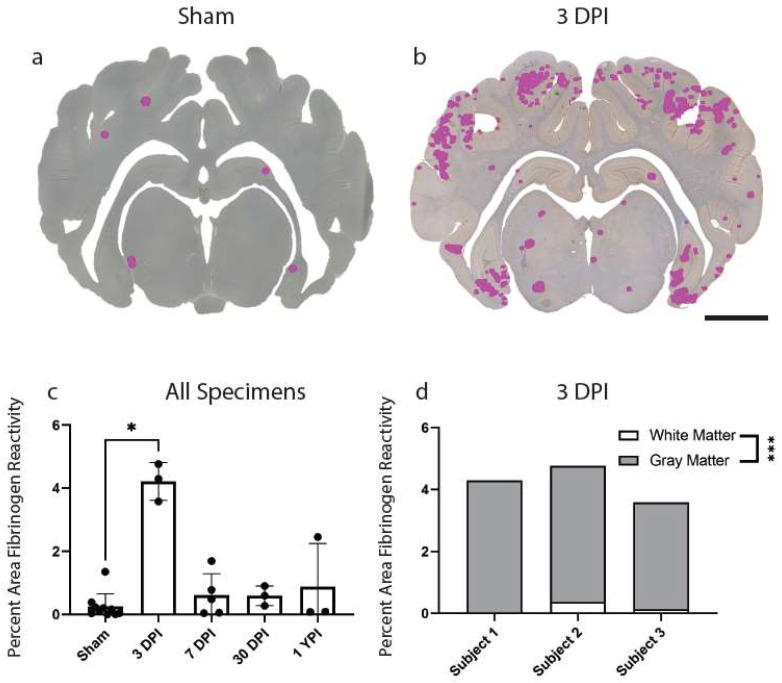
Fibrinogen Reactivity Increases at 3 DPI. Regions of fibrinogen extravasation are shown in sham (**a**) and 3 DPI (**b**) via Stereo Investigator software on whole coronal sections of tissue (scale = 1 cm). The percentage area of fibrinogen reactivity increased at 3 DPI (*p* = 0.0198) compared to sham before resolving by 7 DPI out to 1 YPI (**c**) (* *p* < 0.05). Each point represents data from an individual animal within each group. Fibrinogen annotation in gray versus white matter at 3 DPI demonstrated a statistically significant increase in gray matter (*p* = 0.0003) (**d**) (*** *p* ≤ 0.001).

Lastly, we investigated BBB disruption, a recently appreciated pathological feature of concussion. Extravasation of the serum protein, fibrinogen, has been used to evaluate BBB disruption at acute timepoints following mild TBI in our model of injury and may be an important pathological feature of concussion [31]. Here, we first assessed fibrinogen reactivity out to chronic timepoints in our cohort. There was an overall increase in percentage area of fibrinogen reactivity in the total brain parenchyma (*H* (4) = 10.27, *p* = 0.0360) at 3 DPI (Mean = 4.21, Median = 4.29, SD ± 0.60, 95% CI [2.72, 5.71]) compared to sham (Mean = 0.25, Median = 0.13, SD ± 0.41, 95% CI [−0.04, 0.54]) (*p* = 0.0198). There were no significant changes to percentage area of fibrinogen reactivity at 7 DPI, 30 DPI, and 1 YPI compared to sham (Figure 5c).

Extravasated serum proteins were frequently observed in the gray matter compared to white matter structures at 3 DPI, so we also quantified this gray to white matter reactivity ratio at this timepoint alone (n = 3 per group). There was a statistically significant increase in percentage area of fibrinogen reactivity in gray matter (Mean = 4.04, Median = 4.26, SD ± 0.52, 95% CI [2.75, 5.32]) compared to white matter (Mean = 0.18, Median = 0.13, SD ± 0.18, 95% CI [−0.24, 0.60]) at 3 DPI (t (4) = 12.26, *p* = 0.0003) (Figure 5d).

## 4. Discussion

After a single mild TBI in our pig model of closed-head diffuse brain injury, we found chronic changes in microglial morphology in the cingulate gyrus, as well as acute and subacute changes in microglial morphology in the inferior temporal gyrus. Additionally, we found a significant increase in fibrinogen blood proteins extravasated into the brain at 3 DPI, which resolved to sham levels by 7 DPI. Yet we did not find neuronal loss or astrocyte reactivity paired with these pathological changes. We hypothesized that microglial morphological changes would coincide with astroglial reactivity, neuronal dystrophy/loss, and BBB disruption in the cerebral cortex after TBI. Therefore, we are compelled to reject part of our original hypothesis. These microglia morphological changes supplement and support our previous studies in the white matter and hippocampal subregions, which demonstrated microglia morphological changes, microglia density changes, and axonal pathology after a single mild TBI [9,24].

In the current study, we detected chronically hyper-ramified microglia in cingulate gyrus; however hyper-ramified microglia were only detected at acute and subacute timepoints in inferior temporal gyrus. This discrepancy in pathology may highlight the injury thresholds needed to produce microglial changes after mild TBI in our model. The medial cingulate may be more vulnerable to damage compared to the lateral inferior temporal gyrus at this level of injury, leading to chronic pathology that is consistent along the rostral-caudal axis. Vascular damage or dysfunctional neurons projecting into susceptible white matter may account for this pathology. Additionally, inertial sheer forces in this model of injury may be greater in midline structures versus lateral structures. Follow-up studies are warranted to elucidate the mechanism of region-specific microglial morphological changes.

Absent additional transcriptomic studies, our interpretation of these phenotypic changes on microglia behavior is limited. Cell sorting and RNAseq techniques have highlighted microglia complexity in recent years. Hammond et al. examined individual mouse microglia during development, old age, and after brain injury and detected at least nine transcriptionally distinct microglia subpopulations [32]. Saba et al. characterized several microglia phenotypes following brain injury via multicolor flow cytometry and correlated these subpopulations with long-term cognitive deficits [33]. Furthermore, a 2022 White Paper written by leading microglia researchers urged the field to reject the classic pro- versus anti-inflammatory microglial classifications and provided a new series of recommendations to understand microglial states [34]. Future microglial studies will need to utilize transcriptomic, proteomic, and genetic manipulation techniques to fully characterize microglia subpopulations and determine their functions [35].

Morphological changes in microglia only indicate a change in neuroimmune homeostasis that we are witnessing out to chronic timepoints [9,36]. Importantly, a select few studies have described a hyper-ramified microglia phenotype after injury. After rat midline fluid percussion injury, Morrison et al. conducted microglia skeletal analysis and found a decrease in microglia ramification around the impact site but not in a remote region. Additionally, microglia complexity—measured by fractal analysis—increased in the remote region [37]. Morrison et al. carefully distinguished between ramified microglia and “ramified/hyper-complex” microglia, the former of which is detected most often in sham, while the latter is detected in remote regions at subacute timepoints. Our current findings seem to more closely align with this “hyper-complex” phenotype; our observed microglia have more branches, junctions, endpoints, and summed process lengths that extend far away from the soma. This hyper-ramified phenotype should not be confused with a classic bushy microglia morphology where microglia retract their processes from surrounding tissue and have many short, thickened processes. Finally, studies outside of the TBI literature have reported a hyper-ramified phenotype. One recent study using a mouse model of PTSD reported an increase in hyper-ramified microglia and loss of dendritic spines in specific neuroanatomical regions [38]. Hyper-ramified microglia were also detected in rat prefrontal cortex in a chronic stress model [39]. Overall, microglia becoming de-ramified post-injury is commonly reported while microglia hyper-ramification is observed far less frequently and can indicate stress on the neuroimmune system [37,40].

This hyper-ramified phenotype can be triggered by numerous extracellular signals. For instance, fibrinogen leakage through the BBB is a key change in the extracellular environment that can initiate neuroinflammation (for a review see [41]). Microglia may also be responding to injured neurons and may also be implicated in synaptic modifications [24,40]. Previous research in this model found an acute inflammatory response around permeabilized cortical neurons; microglia density increased and morphology became less ramified 15 min after both mild and severe TBI [23].

Unfortunately, administration of the permeability marker was not performed in this cohort of animals. Yet studies suggest that permeabilized cells initially survive the injury to die at a later timepoint. In particular, Whalen et al. subjected mice to controlled cortical impact and found that many plasma-membrane-damaged neurons recovered their integrity within 24 h, yet disappeared from the brain by 7 DPI [20]. Therefore, we conducted a limited cell density analysis in the cingulate gyrus to determine if neuronal loss coincides with our chronic microglial changes. Neither our total cell density nor our neuronal cell density significantly changed at timepoints post-injury. We initially hypothesized neuronal loss after mild TBI as permeabilized neurons have been prominent in medial gyri and isolated to layers II/III of the cortex [30]. Since neuronal loss was not seen in our cohort, ongoing neuronal dysfunction may be present and potentially driving microglial homeostatic changes and possible microglia-mediated protection. Using a model of cerebral ischemia, Szalay et al. found that microglia reduced excitotoxic damage, as drug-induced knockout of microglia led to dysregulated calcium responses, calcium overload, increased neuronal death, and incidence of spreading depolarization— a process that involves neuronal swelling, injury to dendritic spines, and subsequent dampening of brain electrical activity [42]. Future studies should employ electrophysiological techniques in addition to RNA assays to assess loss of function in cortical neurons in relation to neuroimmune activity.

To further explore the extent of gray matter neuropathology, we utilized other traditional histological markers after TBI. We assessed GFAP reactivity as astrocyte end feet are a critical component of maintaining the BBB, and when disturbed, could promote pathology [43]. We examined both the cingulate gyrus and the inferior temporal gyrus yet did not find any indication of increased astrocyte reactivity after mild TBI. This is consistent with our previous examination of astrocyte reactivity in the white matter [9]. Astrocytes have been documented colocalizing with fibrinogen after mild TBI and reactive astrocytes are considered a hallmark pathology of injured CNS tissue, yet overt astrocyte reactivity does not seem to occur at these injury levels at a level detectable with our methodology [29,31]. Astrocytes may undergo genomic changes in response to injury, so future studies may want to employ RNA-based assays to detect any subtle changes in astrocyte activity post-TBI [44]. Moreover, astrocytes undergo proliferative changes that can vary depending on injury severity so astrocytes should still be examined at higher injury levels or after repetitive mild injuries in this model [44].

We also assessed fibrinogen reactivity as a global measure of blood-brain barrier breakdown and neuropathology post TBI. Previous studies in our injury model have found an increase in fibrinogen after a single mild TBI. Utilizing a different strain of pigs, Johnson et al. (2018) conducted postmortem histology at 6 h and 3 DPI and found an increase in fibrinogen extravasation at both timepoints. They noted fibrinogen was specifically found in gray-white matter boundaries, periventricular, and subpial regions [31]. Our analysis found fibrinogen at gray-white matter boundaries as well as in middle layers of the cortex. The different pig strains and potentially different vasculature may account for the disparities in fibrinogen’s pattern and distribution in the brain parenchyma. Our analysis also found an increase in fibrinogen reactivity at 3 DPI and that this reactivity resolved by 7 DPI out to chronic timepoints. In contrast, Hay et al. (2015) found that 47% of humans that survived a moderate or severe TBI for a year or more showed multifocal parenchymal fibrinogen immunoreactivity [45]. This may suggest that fibrinogen-measured BBB disruption is a common pathological finding after moderate or severe TBI but does not persist beyond the acute phase of mild TBI. Additionally, we examined the extent of fibrinogen reactivity in gray versus white matter at 3 DPI and found a significant presence in gray matter compared to white matter, underscoring the importance of gray matter pathology after mild TBI.

Finally, we found a statistically significant difference in age-matched sham microglia morphology at one year in the inferior temporal gyrus compared to 30-day or less age-matched sham. These differences are specific to the inferior temporal gyrus, which has shown heterogenous reactivity to TBI in our model. This is interesting for future studies in the intersection of aging and TBI, however that is not the focus of this study. Other regions had no detectable differences in sham over time.

There are several limitations in this study. First, the sample size of injured specimens was small, which reduces the power of the study. Large animal subjects are costly and require specialized housing and trained veterinary staff. Additionally, this was an archival study of formalin-fixed paraffin-embedded tissue, which constrained our options for experimental methodology. Also, many commercially available antibodies do not cross-react with pig tissue, thus also restricting our immunohistochemical staining and pathological analysis.

In conclusion, we have detailed a temporal sequence of neuropathology in the cerebral cortex, an often-overlooked area in diffuse brain injury. Fibrinogen-measured BBB disruption increases at 3 DPI but resolves by 7 DPI. Meanwhile, microglia adopt a hyper-ramified morphology from 3 DPI to 30 DPI in the inferior temporal gyrus and are delayed until 30 DPI but persist out to 1 YPI in the cingulate gyrus. These delayed consequences may be an effect of distal diffuse axonal injury and warrant follow-up studies. We have demonstrated that a single, closed-head, mild TBI is associated with chronic alterations to microglia homeostasis. Microglia activity has become increasingly implicated in neurodegenerative disease pathogenesis though precisely how they contribute to disease progression remains elusive [46,47]. Further experimentation is needed to supplement our histological analysis. Microglia modulate synaptic plasticity, therefore presynaptic and postsynaptic markers as well as electrophysiological recordings could examine the impact of hyper-ramified microglia after TBI. Compounds used to transiently knock out microglia should be used in our model to assess potential neuron loss and cell physiological dysfunction that may be microglia-mediated in gray matter. Additionally, neuroimaging would allow us to track microglia activity in a single specimen over time, providing details in between the terminal timepoints in the current study. Finally, no changes in neuronal density were observed but subtle structural and/or functional changes in neurons, axons, and/or dendrites are possible, similar to our recent report of alterations in electrophysiological function absent signs of neuronal or axonal pathology in the hippocampal formation post-TBI in pigs [48]. Golgi staining or dyes used for neuronal tracing can be performed to detect neurostructural changes related to critical functional parameters such as dendritic number, length, and spine density. It is hoped that further understanding of the brain’s inflammatory activity after mild TBI will provide us with knowledge of neuronal health and cognitive integrity, and that this will translate to treatment of TBI in people.

## Figures and Tables

**Table 1:** Skeletal analysis measurements in the anterior cingulate gyrus. All values are reported as mean ± standard deviation, 95% confidence interval [lower 95% CI, upper 95% CI].

	Branches	Junctions	Endpoints	Summed Process Length
Sham	26.66 ± 10.03, 95% CI [23.81, 29.51]	12.87 ± 5.09, 95% CI [11.42, 14.31]	14.56 ± 5.36, 95% CI [13.03, 16.08]	5.82 ± 2.07, 95% CI [5.23, 6.41]
3 DPI	26.51 ± 5.70, 95% CI [23.23, 29.80]	12.73 ± 2.89, 95% CI [11.06, 14.40]	14.11 ± 2.57, 95% CI [12.63, 15.60]	5.75 ± 1.31, 95% CI [5.00, 6.51]
7 DPI	28.69 ± 12.15, 95% CI [23.68, 33.70]	13.83 ± 6.19, 95% CI [11.27, 16.38]	14.91 ± 5.37, 95% CI [12.69, 17.12]	5.66 ± 2.00, 95% CI [4.81, 6.50]
30 DPI	40.77 ± 9.92, 95% CI [35.28, 46.27]	20.00 ± 5.01, 95% CI [17.22, 22.78]	20.33 ± 4.45, 95% CI [17.86, 22.79]	8.91 ± 1.95, 95% CI [7.83, 9.99]
1 YPI	39.49 ± 18.73, 95% CI [29.12, 49.87]	19.41 ± 9.53, 95% CI [14.13, 24.69]	19.40 ± 8.15, 95% CI [14.89, 23.91]	8.09 ± 3.56, 95% CI [6.12, 10.06]

**Table 2:** Skeletal analysis measurements in the posterior cingulate gyrus. All Values are reported as mean ± standard deviation, 95% confidence interval [lower 95% CI, upper 95% CI].

	Branches	Junctions	Endpoints	Summed Process Length
Sham	26.24 ± 8.18, 95% CI [23.92, 28.57]	12.66 ± 4.10, 95% CI [11.49, 13.82]	13.80 ± 3.79, 95% CI [12.72, 14.88]	5.91 ± 1.75, 95% CI [5.41, 6.41]
3 DPI	26.81 ± 12.01, 95% CI [20.16, 33.46]	12.99 ± 6.14, 95% CI [9.59, 16.39]	13.95 ± 5.31, 95% CI [11.01, 16.88]	5.96 ± 2.63, 95% CI [4.50, 7.42]
7 DPI	28.12 ± 11.28, 95% CI [23.46, 32.78]	13.62 ± 5.78, 95% CI [11.23, 16.00]	14.48 ± 4.98, 95% CI [12.42, 16.53]	5.77 ± 2.12, 95% CI [4.89, 6.64]
30 DPI	34.88 ± 11.58, 95% CI [28.47, 41.30]	17.05 ± 5.91, 95% CI [13.78, 20.33]	17.73 ± 5.20, 95% CI [14.85, 20.61]	7.75 ± 2.29, 95% CI [6.48, 9.02]
1 YPI	36.34 ± 14.42, 95% CI [28.35, 44.32]	17.68 ± 7.31, 95% CI [13.63, 21.73]	18.52 ± 6.44, 95% CI [14.95, 22.08]	7.81 ± 2.84, 95% CI [6.24, 9.38]

**Table 3:** Skeletal analysis measurements in the anterior inferior temporal gyrus. All values are reported as mean ± standard deviation, 95% confidence interval [lower 95% CI, upper 95% CI].

	Branches	Junctions	Endpoints	Summed Process Length
Sham	24.94 ± 10.59, 95% CI [21.84, 28.05]	11.54 ± 4.60, 95% CI [10.18, 12.91]	13.19 ± 4.66, 95% CI [11.82, 14.55]	5.07 ± 1.68, 95% CI [4.58, 5.57]
3 DPI	35.52 ± 13.31, 95% CI [28.15, 42.89]	17.30 ± 6.84, 95% CI [13.51, 21.09]	17.98 ± 5.88, 95% CI [14.72, 21.23]	7.34 ± 2.47, 95% CI [5.98, 8.71]
7 DPI	39.39 ± 19.18, 95% CI [31.48, 47.31]	19.16 ± 9.70, 95% CI [15.16, 23.17]	19.68 ± 8.44, 95% CI [16.19, 23.16]	7.11 ± 3.17, 95% CI [5.81, 8.42]
30 DPI	40.99 ± 17.92, 95% CI [31.06, 50.91]	20.04 ± 9.07, 95% CI [15.02, 25.06]	20.55 ± 7.95, 95% CI [16.15, 24.95]	8.01 ± 2.84, 95% CI [6.44, 9.59]
1 YPI	32.80 ± 17.00, 95% CI [23.38, 42.21]	16.06 ± 8.70, 95% CI [11.24, 20.88]	16.47 ± 7.40, 95% CI [12.38, 20.56]	6.91 ± 3.31, 95% CI [5.08, 8.74]

**Table 4:** Skeletal analysis measurements in the posterior inferior temporal gyrus. All values are reported as mean ± standard deviation, 95% confidence interval [lower 95% CI, upper 95% CI].

	Branches	Junctions	Endpoints	Summed Process Length
Sham	26.27 ± 8.09, 95% CI [23.97, 28.57]	12.70 ± 4.11, 95% CI [11.53, 13.87]	13.66 ± 3.63, 95% CI [12.63, 14.69]	5.64 ± 1.64, 95% CI [5.18, 6.11]
3 DPI	44.78 ± 19.86, 95% CI [33.78, 55.78]	21.93 ± 9.94, 95% CI [16.43, 27.44]	22.21 ± 9.11, 95% CI [17.16, 27.25]	8.91 ± 3.62, 95% CI [6.91, 10.91]
7 DPI	40.20 ± 14.75, 95% CI [34.11, 46.28]	19.73 ± 7.35, 95% CI [16.69, 22.76]	19.68 ± 6.57, 95% CI [16.97, 22.39]	7.97 ± 2.92, 95% CI [6.76, 9.17]
30 DPI	37.48 ± 11.68, 95% CI [30.73, 44.22]	18.35 ± 5.95, 95% CI [14.92, 21.79]	18.57 ± 5.02, 95% CI [15.68, 21.47]	7.63 ± 2.31, 95% CI [6.30, 8.96]
1 YPI	34.23 ± 12.09, 95% CI [27.53, 40.93]	16.69 ± 6.18, 95% CI [13.27, 20.11]	17.31 ± 5.24, 95% CI [14.40, 20.21]	7.10 ± 2.26, 95% CI [5.85, 8.35]

**Table 5:** Cohen’s d effect sizes of cingulate gyrus cell counts compared to sham.

	3 DPI	7 DPI	30 DPI	1 YPI
Total Cell Density	*d* = 1.13	*d* = 1.35	*d* = 0.51	*d* = 1.28

## Data Availability

The datasets used during the current study are available from the corresponding author on reasonable request.
